# UNDERSTANDING THE NEEDS AND FUTURE OF OLDER BLACK AND HISPANIC WORKERS IMPACTED BY COVID-19

**DOI:** 10.1093/geroni/igac059.1905

**Published:** 2022-12-20

**Authors:** Kendra Jason

**Affiliations:** University of North Carolina at Charlotte, Charlotte, North Carolina, United States

## Abstract

COVID-19 infection and associated deaths are unequally distributed, as 78.5% of decedents are adults over 65 years of age; and Hispanics accounted for 24.2% of deaths. Black people account for 30% of infections and 18.7% of deaths, although they represent only 12.5% of the population. Older Black and Hispanic adults are in “Double Jeopardy” with their experiences shaped by racism and ageism, thus, putting them at higher risk for exposure to SARS-CoV-2, the virus that causes COVID-19, and poor health outcomes. Centering the intersections of race, age, and socioeconomic status and utilizing a scoping review (N=55), this study identifies the four primary risks faced by Black and Hispanic adults that help explain disparate COVID-19 work outcomes: (1) being an essential worker, (2) type of work performed, (3) workplace risks; and (4) community and geographic risk factors. This study also (1) explores the impacts of COVID-19 influence work participation, and (2) identifies processes linking ageism, racism, health, and employment situations in shaping the health and work-ability of older working adults. This research centers populations in which COVID-19 has had the most devastating financial impact: Black and Hispanic workers, Black women, and low-wage workers. This study increases our understanding of older Black and Hispanic adults lived experiences of managing COVID-19 – information that is critical for planning intervention and support services to ameliorate impact of the disease on Older Black and Hispanic adults; and informs policy and practice for economic recovery from the pandemic for other marginalized populations.

